# Natural Remedies for Irritable Bowel Syndrome: A Comprehensive Review of Herbal-Based Therapies

**DOI:** 10.3390/ijms26199345

**Published:** 2025-09-24

**Authors:** Raquel Abalo, Paula Gallego-Barceló, Daniela Gabbia

**Affiliations:** 1 Department of Basic Health Sciences, University Rey Juan Carlos (URJC), 28922 Alcorcón, Spain; paula.gallego@urjc.es; 2 High Performance Research Group in Physiopathology and Pharmacology of the Digestive System (NeuGut), University Rey Juan Carlos (URJC), 28922 Alcorcón, Spain; 3 R+D+i Associated Unit to the Institute of Medicinal Chemistry (IQM), Spanish National Research Council (CSIC), 28006 Madrid, Spain; 4 Working Group of Basic Sciences on Cannabinoids of the Spanish Pain Society, 28046 Madrid, Spain; 5 Working Group of Basic Sciences on Pain and Analgesia of the Spanish Pain Society, 28046 Madrid, Spain; 6 Department of Pharmaceutical and Pharmacological Sciences, University of Padova, 35131 Padova, Italy

**Keywords:** irritable bowel syndrome, prebiotics, natural compounds, herbal supplements, complementary medicine

## Abstract

Irritable Bowel Syndrome (IBS) is a complex and multifactorial gastrointestinal disorder characterized by recurrent abdominal pain and altered bowel habits, impacting quality of life. Therapy is mainly based on relieving symptoms with specific drugs, whereas herbal and complementary remedies have gained attention in recent years. This review examines the current knowledge on herbal remedies in IBS management. Several herbal treatments, particularly peppermint oil and Iberogast, have demonstrated efficacy in randomized controlled trials. Preclinical studies have revealed promising anti-inflammatory and antispasmodic effects for herbs, e.g., curcumin, fennel oil, and cannabis derivatives. However, many studies suffer from some limitations, e.g., small sample sizes, short study durations, or methodological weaknesses. There is a lack of large-scale, long-term randomized controlled trials for most herbal remedies, and heterogeneity in study designs makes direct comparisons challenging. Moreover, limited evidence exists regarding herb–drug interactions and long-term safety profiles. Despite these limitations, certain herbal remedies may offer a valuable complementary approach for some IBS patients when used under medical supervision. Future research should focus on larger, well-designed clinical trials to establish efficacy, optimal dosing, and long-term safety, as well as elucidate specific mechanisms of action and identify patient subgroups that may benefit most from specific herbal treatments.

## 1. Introduction

Irritable Bowel Syndrome (IBS) represents a complex and multifactorial gastrointestinal (GI) disorder affecting 35 million patients in the United States, with a female–male ratio of 1.8–2:1 [1,2]. Its prevalence is around 11% worldwide, with a significant variability ranging from 1.1% in France and Iran to 35.5% in Mexico, that could reflect differences in geography, culture, and population, but also in diagnostic criteria and study enrolment [3,4]. Although IBS has not been associated with an increase in mortality [5], its symptoms, mainly diarrhea, constipation, and abdominal pain, are debilitating and may strongly impair patients’ quality of life, affecting physical, emotional, and social functions and leading to reduced travel, sex avoidance, and time spent with friends and family [6].

IBS therapy is mainly focused on relieving symptoms with specific treatments, e.g., laxatives, antidiarrheal drugs, rifaximin, and fiber supplements, as well as on managing depressive symptomatology and related stress with neuromodulators and antidepressants [3,7,8]. In recent decades, many patients turn to integrated or complementary approaches to relieve IBS symptoms, including lifestyle modifications, gut-directed hypnotherapy, probiotics, gluten-free diets or diets low in fermentable oligosaccharides, disaccharides, monosaccharides and polyols (FODMAP), and several herbal products [9,10,11,12].

This review aims to illustrate recent advances in the herbal-based remedies for IBS, focusing on the evidence reporting their mechanisms of action, their efficacy in alleviating symptoms and inflammation, and their potential safety concerns. Through this analysis, this review offers an updated perspective on the potential role of natural remedies as adjunctive or alternative treatments for IBS management.

## 2. Pathophysiology of IBS and Diagnostic Criteria

IBS is one of the most prevalent disorders of the gut–brain interaction (DGBI), formerly known as functional GI diseases. These conditions are characterized by the presence of chronic GI symptoms in the absence of identifiable structural or biochemical abnormalities, in contrast to organic GI diseases, which display evident inflammatory features [13].

Since the publication of the first Rome Criteria in 1994, the Rome IV Criteria (the fourth and current iteration) are currently considered to be the gold-standard symptom-based criteria to diagnose DGBI [14]. The Rome IV Criteria define IBS as the presence of persistent or recurrent lower abdominal pain (occurring at least one day per week in the last 3 months), with two or more of the following: related with defecation, associated with a change in stool frequency or associated with a change in stool form. Symptoms must have been present for at least 6 months [15].

Since no universally recognized specific biomarkers exist, diagnosis of IBS is mainly based on the clinical symptoms suffered by the patient, using the Rome IV Criteria. A careful history and physical examination, including digital rectal examination to exclude a rectal mass or dyssynergic defecation are needed when IBS is suspected [16]. Pictograms enhance comprehension by the patient and may be used to more accurately assess symptoms [17]. The IBS symptom severity score (IBS-SSS) is commonly employed to evaluate the severity of the disease, scoring from 0 to 500, with scoring below 175, 175 to 300, and above 300 representing mild, moderate, and severe IBS, respectively [18,19].

Abdominal pain, a hallmark in IBS patients [8,17], is generally self-reported through questionnaires and confirmed by the clinician through detailed medical history and physical examination [17]. In addition to the duration and frequency of abdominal pain events, the visual analog scale (VAS) is often used to evaluate pain intensity, with scores ranging from 0 (no sensation) to 10 (strongest imaginable sensation) [19,20]. Visceral hypersensitivity, a key contributor to abdominal pain in IBS, is defined as the increased perception of visceral stimuli (chemical, mechanical) [21]. Patients with visceral hypersensitivity tend to have abdominal pain, mainly due to intraluminal retention of the gas or solid contents and mechanical stress to the gut wall [22]. As with somatic pain, visceral pain is transmitted to conscious perception in the brain through a 3-neuron pathway, involving peripheral vagal, thoracolumbar, and lumbosacral afferents, which transmit the nociceptive information to the spinal cord nuclei, from which it travels to the upper nuclei in the brain through ascending pathways, with inhibitory modulation exerted by descending pathways. Visceral hypersensitivity may be due to a reduced threshold to the perception of luminal stimuli, to the dysregulation of central processing or both. In IBS, colonic or rectal distension may be used to evaluate the presence of visceral hypersensitivity and could be useful to search for new treatments. Indeed, IBS patients display lowered pain thresholds in response to rectal distension induced by a barostat, as compared to healthy volunteers and patients with other GI diseases [23].

The Bristol scale is used to determine stool appearance, which varies from 1 (hard, lumpy stool) to 7 (watery stool), suggestive of severe constipation and severe diarrhea, respectively, whereas normal stool form is given 3–4 points on this scale [24]. Although IBS is commonly classified into two main subtypes based on stool form and frequency, namely constipation-predominant (IBS-C) and diarrhea-predominant (IBS-D), it is well recognized that many patients experience both constipation and diarrhea in varying proportions, either alternately or simultaneously, a presentation defined as mixed IBS (IBS-M) [14]. According to the Rome Foundation Global Epidemiology Study, IBS-C and IBS-M each account for approximately one-third of all IBS diagnoses, followed by IBS-D (28.7%) and unclassified IBS (6.5%) [4]. Interestingly, GI transit measurement (using radiopaque markers and scintigraphy as the gold standard, as well as other more research-oriented methods such as breath tests, ingestible capsules sensing endoluminal pH or gas content, 3D-GI transit test or magnetic resonance imaging capsule markers test, or even the blue dye muffin or sweetcorn for whole GI transit test) has been proved to be relevant to identify changes in bowel habits (stool frequency and form), although its relevance for other GI symptoms in IBS, such as abdominal pain, is not clear [25].

Importantly, in contrast with organic diseases affecting the bowel such as inflammatory bowel disease (IBD) and colorectal cancer (CRC), imaging and other diagnostic investigations are typically normal in most patients with IBS. Accordingly, as already mentioned, IBS is considered a “functional” disorder, and patients often require reassurance that their symptoms do not indicate a serious underlying disease. Thus, diagnostic investigations such as endoscopy (colonoscopy or flexible sigmoidoscopy, with biopsy), imaging analysis (computed tomography colonography, magnetic resonance enterography, wireless capsule endoscopy) and other diagnostic tools (including routine blood tests, C-reactive protein, celiac serology, fecal calprotectin, fecal elastase, 23-seleno-25-homo-tauor-cholic acid) are sometimes required to exclude conditions that can mimic IBS, such as coeliac disease, IBD, microscopic colitis, CRC or bile acid diarrhea (BAD). Indeed, BAD should be considered as a possible alternative diagnosis in patients with IBS-D [26]. The clinical history needs to also consider the possibility that the recent introduction of a new drug (NSAIDs, PPIs, statins, or selective serotonin reuptake inhibitors, SSRIs) may be causing the symptoms (i.e., diarrhea) [27]. Innovative medical technology, such as confocal endomicroscopy, capsule-based microbiota sampling, and even artificial intelligence approaches, applied to metabolomic profiling and dysbiosis detection, or to the endoscopic detection of subtle disease-associated mucosal changes, is increasingly being used, although still mainly for research, not for daily practice [28].

In addition to the presence of cardinal symptoms, e.g., abdominal pain and changes in bowel habits, and the absence of alarm symptoms and signs of organic disease when these need to be evaluated, IBS diagnosis may be supported by the following features, as determined during clinical history taking: onset post-infection or following recurrent antibiotic use, onset after acute or chronic stress, onset after previous psychological trauma, presence of extraintestinal symptoms, e.g., back pain, tiredness, gynecological issues, presence of other functional non-GI disorders, e.g., fibromyalgia, tension headache, chronic fatigue syndrome, presence of psychological comorbidities, e.g., anxiety or depression [16]. These features are related to the risk factors contributing to IBS development, as described in depth below, and its common comorbidities.

Despite performing better in terms of specificity, Rome IV criteria are somehow restrictive and less sensitive than Rome III (the previous iteration of the Rome criteria), although, interestingly, this can be improved by relaxing the number of days with abdominal pain to 3 per month instead of 1 per week [26], and this has been suggested as a possible improvement of future Rome Criteria iterations [26,29]. Whatever the case may be, using symptom-based criteria properly is key to avoid over-investigation, which, in addition to being generally unnecessary, as well as costly and time-consuming for the health system, may cause anxiety in patients with IBS [16]. This allows making a positive diagnosis of IBS and establishing treatment measures to manage it. For this, it is important first to explain the etiology and natural history of the condition in the context of the gut–brain axis to the patients, to manage their expectations (explaining that there is no cure for IBS and that long-term treatment is likely to be necessary), to provide lifestyle and dietary advice, including the use of probiotics or a low FODMAP diet in some cases, and, if no improvement of symptoms is achieved, to discuss treatment options targeted at the predominant symptom [16], as follows:

- Abdominal pain: antispasmodics (hyoscine, peppermint oil) and gut–brain neuromodulators (e.g., a tricyclic antidepressant) as first- and second-line treatments, respectively,
- Constipation: laxatives and secretagogues (lubiprostone, linaclotide), as first- and second-line treatments, respectively,
- Diarrhea: loperamide and 5-HT3 receptor antagonists, eluxadoline or rifaximine, as first- and second-line treatments, respectively.

For refractory symptoms, brain–gut behavioral treatments, such as IBS-specific cognitive behavioral therapy or gut-directed hypnotherapy are suggested [16]. These approaches are primarily designed to treat GI symptoms, rather than to address psychological health needs per se, although mental health is recognized to be an important factor contributing to maintaining GI symptoms in IBS. Indeed, anxiety or depression symptoms affect 20–40% IBS patients [30] and somatization scores are elevated in them [31].

Thus, being a DGBI, accurate diagnosis of IBS should consider not only the GI symptoms but also its psychological impact, and recent studies suggest that IBS may be subclassified in at least seven clusters [32], as shown in Table 1.

These clusters are important because they predict healthcare-seeking, prior abdominal surgery, symptom severity, and impairment in quality of life in people with IBS [32]. Moreover, treatment can be adjusted according to the cluster to which the patient belongs. Thus, patients with low levels of GI and psychological symptoms (i.e., cluster 3) could be managed with simple dietary advice, first-line drugs, or reassurance. Individuals with predominantly GI symptoms (i.e., clusters 1 and 7) could be treated just with peripherally acting drugs (antidiarrheals, laxatives) targeting those specific symptoms. Patients with mainly abdominal pain and psychological symptoms (i.e., cluster 2) may benefit from a brain–gut behavioral therapy. Moreover, in patients with a combination of GI and psychological symptoms (i.e., clusters 4 and 5), combination therapies with a peripherally acting drug and either a gut–brain neuromodulator or brain–gut behavioral therapy may be the best therapeutic option. In patients with a high prevalence of other chronic medical conditions and previous abdominal surgery (i.e., cluster 6), a multidisciplinary approach including psychological input would be required. These findings may also be useful for the formulation of the coming Rome V Criteria.

It is important to highlight that the current concept of IBS, through Rome IV evolving to Rome V diagnostic criteria (to be released in 2026, [29]) recognizes the complex interplay between the neuroendocrine axis, gut microbiota, inflammatory markers, and GI symptoms, leading for the first time to the application of multiparametric approaches to study IBS, in accordance with the biopsychosocial model [33,34]. Thus, in a recent study conducted in IBS patients (irrespective of their subtype) in comparison with controls, the levels of adrenocorticotropic hormone (ACTH), cortisol, and serotonin were higher, *Bacteroides* were more prevalent and *Bifidobacteria*, *Lactobacillus*, and *Clostridium* were less prevalent, and pro-inflammatory IL-6 and IL-8 were higher, whereas the anti-inflammatory cytokine IL-10 was lower. Interestingly, ACTH and cortisol increases were greater in IBS-D than in IBS-C, irrespective of the measurement point (8 a.m., 4 p.m., 12 a.m.) [33]. Importantly, other researchers have recently used functional magnetic resonance imaging (fMRI) and the functional connectivity strength (FCS) method together with machine-learning algorithms to differentiate between IBS patients and healthy controls, revealing a unique pattern of FCS alterations in brain areas involved in pain regulation and emotional processing. These findings suggest that the identified abnormal FCS features might serve as effective biomarkers for IBS classification [19].

Various risk factors, such as mental stress, mainly in childhood, a previous transient infection or chronic low-grade inflammation of the GI tract, abnormal intestinal microbiota (dysbiosis), and dysfunction of colon motility have all been related to the development of IBS symptoms [35,36]. In these regards, Sulaimi and collaborators recently performed a study on the risk factors for developing IBS and found eight main categories (with some overlaps): dietary, genetic, environmental, psychological, gut microbiome, socio-economic, physiological, and pathological [37]. Among the different risk factors identified, the authors showed that the most frequently reported ones were female gender, gastroenteritis, anxiety disorders, and depression [37]. By identifying the risk factors underlying the onset of IBS, preventative measures can be established.

The gender-dependent differences in IBS incidence, prevalence, severity, and somatic comorbidities, such as joint and muscle pain, are related with the modulatory effect of progesterone on the serotonin system controlling peristalsis; furthermore, estrogen and progesterone inhibit smooth muscle contraction, contributing to explaining the reason for the higher incidence of the IBS-C type in women compared to men [38,39]. In a recent study, female patients showed extensive microstructural alterations in sensorimotor, corticothalamic, and basal ganglia circuits involved in pain processing and the integration of sensorimotor information, suggesting that the etiology and maintenance of symptoms for females with IBS is driven by greater central sensitivity for multiple sensory stimuli [40]. Interestingly, the proportions of female individuals were greatest in clusters with the highest psychological burden (Table 1) in the previously mentioned study [32].

Regarding gastroenteritis, different pathogens have been identified to cause post-infectious IBS (PI-IBS), such as *Norovirus*, *Rotavirus*, *E. coli*, *Salmonella* spp., *Campylobacter* spp., *Giardia duodenalis* and, more recently, SARS-CoV-2 [37,41]. PI-IBS is probably due to prolonged imbalances in the host immune cells and mediators affecting inflammatory homeostasis, as well as disruption of the intestinal mucosal barrier and intestinal dysbiosis, favoring the hypersensitization of nerve afferents [42,43]. The changes in microbiota composition caused by gastroenteritis, antibiotic use, food selections, and stress underlying the development of IBS, justify the recent interest in evaluating the therapeutic potential of probiotics in clinical trials. Additionally, prebiotics may also be useful to preserve a healthy microbiota balance [11,28].

The impact of stress on the development of IBS and the exacerbation of its symptoms is associated with the presence of anxiety and/or depression in these patients through the hyperactivation of the hypothalamic–pituitary–adrenal (HPA) axis and sympathetic nervous system, leading to altered gut motility and increased visceral sensitivity [44]. Microbiota dysbiosis is also induced by stress, facilitating gut barrier dysfunction (“leaky gut”) and immune system activation, a low-grade chronic inflammation contributing to dysmotility [45]. Failure in IBS symptom resolution in patients with undiagnosed anxiety and depression may facilitate seeking alternative therapies, whereas correct pharmacotherapies and psychotherapies may suffice [16].

All these studies highlight the complexity of IBS and the difficulties in realizing an accurate diagnosis and finding fully satisfactory treatments, which must address the different symptoms and signs of IBS in a personalized manner, ideally based on the pathogenetic mechanisms [16,46].

**Figure 1:**
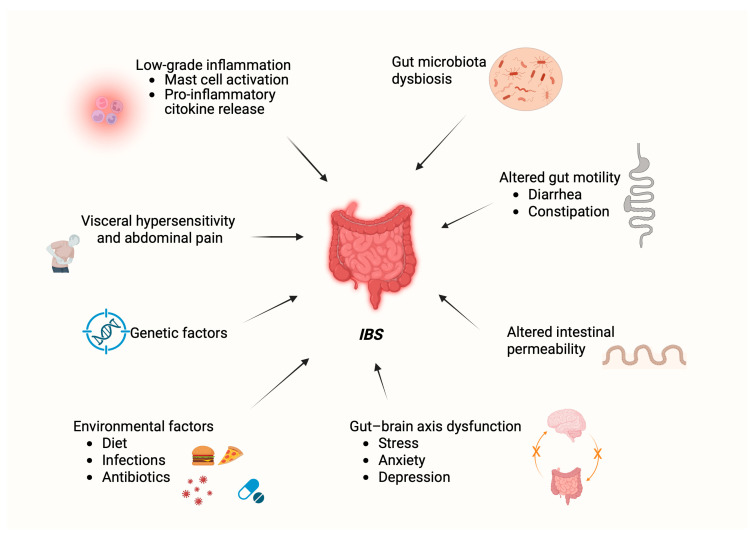
Main pathogenetic features and risk factors contributing to Irritable Bowel Syndrome (IBS). Created in BioRender. Gabbia, D. (2025) https://BioRender.com/ir12zjv.

Indeed, IBS is a multifactorial disease involving multiple pathogenetic mechanisms intricately interconnected, operating in a complex synergistic manner (Figure 1). Gut microbiota dysbiosis can alter intestinal permeability by producing metabolites that affect tight junction proteins, which may trigger low-grade inflammation as bacterial products that may interact with immune cells in the lamina propria [47]. This persistent low-grade inflammatory state, in which mast cells seem to exert a key role [48], may trigger visceral afferent neurons, contributing to visceral hypersensitivity [49]. The modulation of transient receptor potential (TRP) channels, particularly TRPM8, TRPA1, and TRPV1, plays a key role in this process, influencing both pain perception and gut motility [50,51]. Meanwhile, stress-induced changes in the gut–brain axis, mediated via the HPA axis and autonomic nervous system, can alter neurotransmitter release and smooth muscle function, which is able to modulate gut microbiota, thus creating a feedback loop [49]. The activation of nuclear factor kappa B (NF-κB) pathways induced by leaky gut further fuels inflammation by regulating cytokine production and cellular stress responses [52,53]. Genetic factors may predispose individuals to these disturbances, affecting serotonin signaling, immune responses, and overall gut homeostasis. Additionally, the endocannabinoid system, particularly through the activation of CB1, CB2, and TRPV1 receptors, modulates inflammation, pain perception, and motility [54,55]. Oxidative stress, resulting from low-grade inflammation and metabolic changes, can damage the intestinal epithelium, exacerbate permeability issues, and impair smooth muscle function [56].

The complexity of IBS pathogenesis requires a multi-targeted therapeutic approach, which may simultaneously modulate several molecular targets to address multiple aspects of this disorder [46]. Developing new drugs for IBS is also difficult because of the lack of biomarkers and endpoints, which implies high placebo response rates, which do not facilitate pharmaceutical companies’ investment in this field [57,58,59]. The few new drug classes that have been developed recently have not led to any incremental increase in the likelihood of therapy success [60].

In recent years, there has been an increased acceptance of medicinal plants, driven by the belief that natural products have fewer side effects and are more effective and more affordable than their synthetic counterparts [11,12]. Given the complex and multifactorial nature of IBS, these herbal-based therapies have emerged as promising therapeutic and complementary approaches to conventional treatments, e.g., peppermint oil has already been mentioned as conventional antispasmodic first-line treatment of abdominal pain. The following section will explore in detail the mechanisms of action, clinical evidence, and potential benefits of various herbal remedies in IBS management.

## 3. Herbal-Based Remedies for IBS Management

Herbal-based remedies have gained significant attention as complementary treatment options to relieve IBS symptoms, in addition to lifestyle modifications, probiotics, gluten-free diets or diets low in FODMAPs [9,10,11,12]. These herbal-based remedies or natural-derived molecules are of particular interest due to their potential to address multiple aspects of IBS pathophysiology simultaneously. Many herbal compounds exhibit properties that can modulate intestinal motility, reduce inflammation, and alleviate visceral pain, thus potentially offering a more holistic approach to symptom management. Moreover, the generally favorable safety profiles of many herbal remedies make them attractive options for patients seeking alternatives or adjuncts to standard pharmacological interventions. In this section, various herbal treatments showing efficacy in alleviating IBS symptoms are discussed on the basis of literature data. Natural remedies, derived from plants with long histories in traditional medicine, e.g., Chinese herbal medicine, offer potential alternatives or complementary approaches to conventional IBS therapies.

This section covers a range of herbal options, including peppermint oil, Iberogast (STW 5), curcumin, fennel essential oil, ginger, *Aloe vera*, *Cannabis sativa*, and other less-studied herbs (Table 2). To provide a state-of-the-art report of these studies, an analysis of the literature published in PubMed, Scopus, and WOS up to July 2025 was performed using as searching terms “IBS”, “herbal remedies”, and/or the specific name of the herbal remedies, e.g., peppermint oil and others. Each subsection includes a description of the main bioactive compounds responsible for biological effects, proposed mechanisms of action, and available clinical evidence supporting their use in IBS management. By examining these herbal remedies, we aim to provide a comprehensive overview of their potential benefits, limitations, and current state of research in the context of IBS treatment.

### 3.1. Peppermint Oil

Peppermint oil, extracted from the perennial flowering plant *Mentha piperita*, represents one of the most investigated herbal remedies for IBS. Menthol is the primary bioactive component, while other components include limonene, menthofuran, a-pinene, b-pinene, neomenthol, isomenthone, and 1,8-cineole.

Menthol is able to inhibit smooth muscle contractions through the blockade of calcium channels [61,62], thus relieving colonic spasms. However, other evidence suggested that menthol could act on interstitial cells of Cajal (ICCs), the pacemaker cells responsible for slow waves in GI smooth muscle, modulating the activation of transient receptor potential cation channel, subfamily A, member 1 (TRPA1), inducing membrane potential depolarization in the murine small intestine, thus explaining the excitatory effect of menthol on GI motility observed in some studies [63]. As suggested, TRPA1-mediated depolarization of ICCs does not necessarily result in an overall increase in smooth muscle contractility, rather, this study helps in our understanding of the multiple targets of menthol in the GI tract, suggesting that its net effect derives from the balance between inhibitory pathways, e.g., Ca^2+^ channel blockade and transient receptor potential cation channel subfamily M member 8 (TRPM8) activation, and excitatory pathways, e.g., TRPA1, Rho-kinase, and COX activation [63,64,65].

Menthol also displays anti-inflammatory, anti-microbial, and anti-fungal properties that could be useful in modulating immune system function and pro-inflammatory cytokine levels, improving IBS-related inflammation, and microbiota composition, whose dysregulation is reported in IBS patients [66,67,68].

The above-mentioned multiple mechanisms of action support menthol therapeutic potential in IBS. Multiple randomized controlled trials have demonstrated the efficacy of peppermint oil in alleviating IBS symptoms, suggesting its use as a promising treatment option for this condition [69]. A recent phase 3 trial assessing the efficacy of an oral capsule of ZO-Y60 (containing 187 mg of peppermint oil) observed an improvement of the patient’s global assessment (PtGA), IBS symptom severity score, stool frequency score, and stool form score in 69 Japanese patients, corroborating the findings of the previous clinical studies in Europe and the US (JAPIC Clinical Trials Information number: JapicCTI-121727) [70].

### 3.2. Iberogast (STW 5)

Iberogast (STW 5) is a complex herbal preparation developed in Germany in 1961 with nine plant extracts of bitter candytuft (*Iberis amara*), angelica root (*Angelicae radix*), milk thistle fruit (*Silybi mariani fructus*), celandine herb (*Chelidonium majus*), peppermint herb (*Menthae piperitae folium*), caraway fruit (*Carvi fructus*), liquorice root (*Liquiritiae radix*), balm leaf (*Melissae folium*), and chamomile flower (*Matricariae flos*) for treating dyspepsia and irritable colon [71]. Iberogast has been proposed to treat IBS, particularly in the pediatric population, due to its multiple effects. It promotes the relaxation of smooth muscles of the GI tract exerting a spasmolytic effect, stimulates gastric secretions and bile release, and reduces visceral hypersensitivity by virtue of its multi-target composition [72,73]. It has also been demonstrated that Iberogast displays anti-inflammatory properties, by inhibiting the expression of inflammatory mediators NF-κB, STAT1, and iNOS, and counteracting the cytokine-mediated downregulation of ZO-1, a protein involved in tight junction disruption [74]. A prospective observational study evaluating STW-5 therapy (10–20 drops three times daily) in 418 IBS pediatric patients (age 3–14 years) observed an improvement of GI symptoms with a decrease from 19.1 ± 8.9 to 4.8 ± 4.6 of the GI symptom score and a good tolerability and only 0.7% patients reporting adverse events [75]. In a cross-over study with 10 IBS patients with bloating, two-week treatment with STW5 was demonstrated to improve colonic gas tolerance during a gas challenge test, with benefit in treating gas-related abdominal symptoms (EudraCT: 2019-003976-38, www.clinicaltrialsregister.eu) [76].

### 3.3. Curcumin

Curcumin, the major active constituent of *Curcuma longa*, is well known for its potent anti-inflammatory and antioxidant effects [77]. It has also been demonstrated that curcumin could modulate the levels of 5-hydroxytryptamine (5-HT), increasing those in the hippocampus and decreasing those in the colon of IBS rats [78]. This neurotransmitter plays a major role in controlling GI motility, pain perception, and inflammatory responses, and abnormal gut–brain axis has been identified as a risk factor for IBS development [79]. A study assessing the effects of curcumin in a rat model of IBS-C observed that its supplementation improved intestinal motility, altered gut microbiota composition, and reduced the serum levels of 5-HT, substance P, and vasoactive intestinal peptide [77]. Thus, the modulation of gut microbiota and neurotransmitter levels are two key mechanisms of curcumin effects on IBS. Since curcumin has low absorption and bioavailability, it remains unclear whether its effect is primarily due to the modulation of gut microbiota, which in turn modulates neurotransmitter levels, or if it directly impacts the nervous system after absorption.

A recently published systematic analysis of 26 studies reviewing the evidence on the efficacy and safety of curcumin in digestive disorders demonstrated that it is potentially effective for IBS, although most of the reported studies have a high risk of bias and methodological limitations [80]. Thus, the authors concluded that well-designed, large-scale trials are needed to draw robust conclusions about the efficacy of curcumin-based regimens in digestive conditions like IBS.

### 3.4. Fennel Essential Oil

Fennel (*Foeniculum vulgare*) is a perennial plant with a long history of use in traditional medicine. The main bioactive components extracted from its seeds are anethole, fenchone, and estragole, whose antispasmodic activity was observed on tracheal smooth muscle [81]. Anethole is a phytoestrogen structurally similar to catecholamines, like dopamine, and is known for its relaxing effect on intestinal smooth muscle through the activation of potassium channels, leading to membrane hyperpolarization and reduced excitability of smooth muscle cells. Other phenolic chemicals of fennel essential oil, such as flavonoids, may contribute to its positive effect on IBS due to their anti-inflammatory effects, modulatory effect of pro-inflammatory cytokine release, and of gut motility, and attenuation of visceral hypersensitivity, thus exerting a muscle-relaxing action [104].

Fennel oil is widely recognized for its relieving effects on infantile colic and digestive problems [82], but it could also be effective for the treatment of IBS symptoms, frequently in combination with medicinal plant ingredients [105]. A randomized, placebo-controlled trial assessing the efficacy and tolerability of a combination of curcumin and fennel essential oil, named CU-FEO, in 121 patients with mild-to-moderate IBS observed a reduction in abdominal pain and other IBS symptoms, assessed as a reduction in IBS-SSS, as well as an improvement of general quality of life after 30 days of treatment [83]. These results confirm a previous “real life” study on 211 patients with IBS-D, IBS-C or IBS-M enrolled by general practitioners that observed a decrease in IBS severity index and enhanced quality of life across all IBS subtypes [84]. In conclusion, fennel essential oil and its bioactive components may alleviate IBS symptoms by relaxing intestinal smooth muscle, reducing visceral hypersensitivity through anti-inflammatory and neuro-modulatory effects, and promoting a healthier gut microbiota balance [83].

### 3.5. Ginger

Ginger, derived from the rhizome of *Zingiber officinale*, has been used for thousands of years for the treatment of various GI issues, e.g., nausea, vomiting, and dyspepsia. Ginger efficacy in IBS treatment is debated. A study reported that saponins, flavonoids, and alkaloids present in the ginger crude extract exhibit spasmolytic and spasmogenic effects due to the inhibition of cholinergic response and K^+^-induced contractions [85]. In a rat model of IBS-D, ginger extract, particularly its main component 6-gingerol, was able to significantly reduce colonic edema and inflammation, due to the NF-κB-dependent modulation of pro-inflammatory factors, showing effects comparable to rifaximin in improving IBS symptoms [86]. At variance, a pilot double-blind randomized controlled trial enrolling 45 IBS patients failed to observe any difference between the group treated with ginger (1 g or 2 g daily for 28 days) and placebo group in reducing IBS symptoms or providing adequate relief [87]. Another clinical trial in 60 patients with mild-to-moderate IBS symptoms observed that the administration of a herbal mixture containing *Zingiber officinale*, *Boswellia carterii*, and *Achillea millefolium* decreased abdominal pain severity and frequency, bloating, depression, and anxiety scores, accompanied with a significant improvement of quality of life in male patients compared with placebo [88].

### 3.6. Aloe vera

*Aloe vera*, a plant from the *Liliaceae* family, is widely used in traditional medicine as a laxative due to its main component, barbaloin. Once metabolized into aloe-emodine-9-anthrone by intestinal bacteria, it increases intestinal motility and paracellular permeability through the inhibition of Na^+^/K^+^-adenosine triphosphatase in colonic mucosa cells, stimulates mucus secretion and the release of prostaglandin-like compounds in the colon [89]. These actions result in increased water content in the intestinal lumen, producing a laxative effect that has been exploited for constipation treatment in IBS adults, although concerns about potential long-term side effects have been raised [90]. In general, the use of *Aloe vera* is not recommended in pediatric populations [89]. A pooled analysis of two controlled studies suggests that the treatment with *Aloe barbadensis* extract significantly reduces symptom severity and improves response rates in IBS-D patients primarily by reducing abdominal pain severity and frequency, even though the mechanisms underlying these effects remain unclear [91].

### 3.7. Cannabis sativa

*Cannabis sativa*, commonly known as cannabis, mainly produces two well-known phytocannabinoids: Δ^9^-tetrahydrocannabinol (THC) and cannabidiol (CBD). It also contains smaller amounts of minor cannabinoids, like cannabigerol (CBG), and the terpene β-caryophyllene. Together, these compounds act primarily through CB_1_ and CB_2_ receptors and influence TRP ion channels and peroxisome proliferator-activated receptor (PPAR-) α/γ. These pathways help regulate gut motility, low-grade inflammation, and visceral pain, which are central processes altered in IBS.

Preclinical evidence supports its therapeutic potential. Using MFF, a crude ethanolic extract of whole cannabis flower with negligible systemic THC, researchers showed it could block gastric-distension pain and speed colitis recovery in rats through CB_2_ activation [92]. In the DSS colitis model, a CBG-rich hemp oil reduced disease activity, preserved colon length, and restored microbiota balance [93], while β-caryophyllene lowered inflammatory cytokines and visceromotor responses through CB_2_ and PPAR-γ signaling [94]. These findings highlight a peripheral, non-psychoactive pathway that simultaneously addresses inflammation, abnormal transit, and visceral pain. Furthermore, a recent study investigating a variety of cannabinoids in an experimental model of colitis demonstrated that non-psychotropic cannabinoids may exert an abdominal antinociceptive synergistic (“entourage”) effect acting on voltage-gated Na^+^ and Ca^2+^ channels [95].

Early human evidence, although still limited, aligns well with these preclinical signals and shows how natural *Cannabis sativa* compounds may influence multiple IBS symptoms beyond inflammation alone. In IBS-D and IBS-A, a single 2.5–5 mg oral dose of dronabinol (pure Δ^9^-tetrahydrocannabinol, chemically identical to the THC produced by *Cannabis sativa*) slowed fasting colonic transit and increased compliance in the colon, supporting motility control and a higher visceral pain threshold, especially in individuals with favorable *CNR1* or *FAAH* genotypes [96]. However, not all findings are consistent. In another study, a single 8 mg oral dose of THC did not affect visceral sensitivity to distension in the rectum of patients with mixed-type IBS (IBS-M) or healthy volunteers, suggesting that the effect may depend on the specific gut region studied (colon versus rectum), the IBS subtype, baseline visceral hypersensitivity, or differences in dose and study design [97]. CBD by itself remains modest in effect; in an unstratified crossover study using 50 mg CBD chewing gum as needed, reductions in visceral pain and bloating were only numerical and did not reach significance, except in the cluster of patients with moderate-to-severe pain [98]. Real-world experience with whole-plant preparations adds further support, as six months of daily use of THC-rich *Cannabis sativa* flower (Bedrocan^®^, 22% THC: 1% CBD) was linked to sustained improvements not only in visceral pain but also in bloating and stool-form stability in patients meeting Rome IV IBS criteria [99], though the lack of a control arm still limits definitive conclusions. 

In summary, natural compounds from *Cannabis sativa* show promise for addressing dysmotility, low-grade inflammation, and visceral pain in IBS. However, human data remain limited and larger controlled studies are needed to confirm their safety and efficacy.

### 3.8. Coffee

Coffee consumption, widely prevalent worldwide, has been investigated in relation to IBS, showing heterogeneous results that suggest possible preventive effects but limited evidence of symptomatic benefits in diagnosed patients.

In the general population without IBS, a recent meta-analysis including more than 430,000 individuals reported that regular coffee intake was associated with a lower likelihood of developing IBS (OR: 0.84), possibly due to the antioxidant and anti-inflammatory effects of its bioactive compounds (polyphenols, diterpenes, melanoidins), favorable modulation of the gut microbiota, and enhancement of intestinal barrier integrity [100]. However, this study did not evaluate improvements in GI symptoms in patients already diagnosed with IBS, nor did it analyze sex- or subtype-specific effects. Among individuals with existing IBS, available evidence mainly points to an aggravating role of coffee. An analysis of 9710 symptom and dietary diaries showed that coffee, particularly caffeinated coffee, was acutely associated with episodes of diarrhea occurring within 1–2 h post-consumption; although decaffeinated coffee could also trigger symptoms, its effect was less pronounced [101]. These findings suggest that both caffeine and other coffee compounds may stimulate intestinal motility, increase gastric secretion, and trigger mediators such as gastrin, promoting urgency and worsening abdominal pain. Additionally, a cross-sectional study in an Iranian population found that consuming coffee at least weekly increased the likelihood of having IBS by 44%, and high caffeine intake (≥106.5 mg/day) raised the risk by 47%. This study analyzed sex and IBS subtype differences, revealing that the association was significant only in women (OR: 1.48; 95% CI: 1.10–2.00), whereas no clear relationship was observed in men [102]. Moreover, overweight or obese individuals showed a 72% higher likelihood of IBS with high caffeine consumption (OR: 1.72), along with greater symptom severity. Specifically, elevated coffee and caffeine intake were associated with a higher prevalence of constipation-predominant IBS (IBS-C) (OR: 1.66 for coffee; OR: 1.49 for caffeine), suggesting a detrimental rather than a therapeutic effect. Regarding clinical benefits in diagnosed IBS patients, the evidence is very limited: while coffee has a demonstrated a stimulatory effect on colonic motility [103], no randomized controlled trials have shown sustained improvement in symptoms such as constipation, abdominal pain, or bloating in IBS patients [106].

It is important to note that studies on coffee and IBS remain scarce, mostly observational, and heterogeneous in design, coffee type, dose, and diagnostic criteria, with limited stratification by sex and IBS subtype. No randomized clinical trials have directly assessed the impact of coffee on IBS symptoms, making it difficult to draw firm conclusions. Considering the evidence, while coffee may help protect against IBS in healthy individuals [100], in diagnosed patients, especially women, overweight individuals and those with IBS-C, it may precipitate and aggravate symptoms, particularly diarrhea and abdominal pain [101,102]. These findings suggest that coffee intake recommendations for IBS should be personalized and cautious due to limited evidence and possible symptom aggravation.

### 3.9. Yellow gentian

Another herb well known for its potential antispasmodic effects on the digestive system is yellow gentian (*Gentiana lutea*) [104]. Its spasmolytic action is attributed to several bitter bioactive compounds, including gentiopicroside, gentisin, and swertiamarin, that could relax smooth muscle tissue and improve GI function. These compounds stimulate the production of bile and digestive enzymes, potentially enhancing digestive processes and alleviating symptoms, such as cramping and bloating. Through these mechanisms, *Gentiana lutea* may offer therapeutic benefits for IBS symptoms, even though no specific studies have been reported in the literature [104].

### 3.10. Chinese Herbal Medicine

Chinese herbal medicine, particularly standardized multi-herb formulas, has gained attention as a potential complementary therapy for IBS. Chinese herbal medicine formulas are very variable and typically include *Paeonia lactiflora*, *Atractylodes macrocephala*, and *Citrus Reticulatae Blanco* [107]. Tong-Xie-Yao-Fang and its variations are the most tested Chinese herbal preparation for IBS. It consists of four herbal components: *Radix Paeoniae Alba* (Baishao), *Pericarpium Citri Reticulate* (Chenpi), *Radix Saposhnikoviae* (Fangfeng), and *Rhizoma Atractylodis Macrocephalae* (Baizhu) [108]. Preclinical studies suggest that these herbal combinations may act through multiple mechanisms playing a role in IBS pathogenesis [109]. They may reduce mast cell-mediated visceral hypersensitivity, modulate brain–gut axis mediators (e.g., serotonin, corticotropin-releasing factor, BDNF), inhibit colonic smooth muscle hypercontractility, and restore a physiological gut microbiota, leading to decreased mucosal serotonin. One of the bioactive compounds of many Chinese remedies is quercetin, that has been demonstrated to alleviate inflammatory IBS by lowering 5-HT levels, modulating enteroendocrine cell differentiation, and reducing visceral hypersensitivity [110].

A recent meta-analysis of randomized controlled trials, involving approximately 2500 participants, showed that Chinese herbal medicine was more effective than placebo in relieving global IBS symptoms and abdominal pain. Nevertheless, in many studies on IBS, the Chinese herbal medicines used are often insufficiently characterized, which may limit reproducibility and a comprehensive understanding of their therapeutic effects. Reported adverse events, while more frequent than with placebo, were generally mild and primarily affecting the GI tract. Chinese herbal medicine shows promise for short-term symptom relief in IBS, particularly IBS-D; however, many clinical trials included only a limited number of subjects, thus limiting the strength and the relevance of the observations [107]. Further well-designed trials are needed to confirm the long-term efficacy and safety of Chinese herbal medicine and to establish standardized formulations.

## 4. Conclusions and Future Directions

IBS is a common multifactorial GI disorder characterized by recurrent abdominal pain associated with changes in bowel habits, affecting millions of people worldwide and significantly impairing their quality of life [3]. The currently available pharmacological options include drugs treating specific symptoms, such as secretagogues, gut motility modulators, antibiotics, and receptor-targeting agents, and the therapy is personalized according to the subtype of IBS (constipation-predominant IBS-C, diarrhea-predominant IBS-D, or mixed) [8]. In recent years, integrated or complementary approaches, such as lifestyle changes, probiotics, gluten-free and low-FODMAP diets have been suggested for IBS treatment. Furthermore, various herbal-based remedies, many of them with a long history of use for GI symptoms, are currently attracting a growing interest due to increasing evidence on their potential to alleviate symptoms, reduce inflammation, and offer a safe adjunct or alternative treatment option for IBS management [11,12,111]. Table 3 shows a summary of the main clinical studies reporting clinical outcomes observed in IBS patients.

Several herbal treatments, particularly peppermint oil, have demonstrated efficacy in multiple randomized controlled trials, providing a relatively robust rationale for their use in IBS management. Iberogast (STW 5), which combines multiple herbs, has demonstrated benefits in large observational and clinical studies, suggesting its effectiveness as complementary or integrative treatment for symptom relief in IBS patients [112]. Preclinical studies have revealed promising anti-inflammatory and antispasmodic effects for various herbs, including curcumin, fennel oil, and cannabis derivatives. Additionally, certain herbal treatments, like combinations of curcumin and fennel oil, have been demonstrated to induce improvements in validated scores assessing IBS symptoms and quality of life. Likewise, some Chinese herbal medicines, which include different combinations of plants, thereby addressing different symptoms through different mechanisms of action, have also demonstrated usefulness in IBS treatment.

On the other hand, the current knowledge on some of these herbal-based treatments suffers from significant limitations. For some herbal remedies, only preclinical evidence is available, whereas many clinical studies have small sample sizes, short follow-up periods, heterogeneous designs, and outcome measures that complicate direct comparisons of different herbal treatments and could affect the robustness of their conclusions [113]. The heterogeneity in study designs, dosing regimens, and outcome measures make direct comparisons of efficacy between different herbal treatments challenging. For many herbal remedies, the specific mechanisms of action and optimal dosing in the context of IBS remain poorly understood. There is also limited evidence regarding potential herb–drug interactions and long-term safety profiles for the extended use of these treatments. Most studies have not examined differential effects across IBS subtypes or patient subgroups, limiting our understanding of which patients might benefit most from specific herbal interventions.

Despite these limitations, the current evidence suggests that certain herbal remedies, especially peppermint oil and Iberogast (STW 5), may offer a valuable complementary approach for some patients with IBS. However, larger and better-designed clinical trials are warranted to more definitively establish efficacy, optimal dosing, and long-term safety. Future research should also aim to elucidate specific mechanisms of action and identify which patient subgroups may gain more benefit from each herbal treatment. This will help to refine the use of herbal remedies in IBS management and potentially lead to more targeted and effective treatment strategies.

## Figures and Tables

**Table 1:** Subclassification of IBS patients in clusters, according to recent studies that take into consideration IBS’ psychological impact and not only GI symptoms.

Cluster (%) ^1^	GI Symptoms	Psychological Burden
1 (6%)	Diarrhea and urgency	Low
2 (33%)	Low bowel symptom severity with abdominal pain	High
3 (36%)	Low overall GI symptom severity	Low
4 (10%)	Diarrhea, abdominal pain, and urgency	High
5 (3%)	Constipation, abdominal pain, and bloating	High
6 (4%)	High overall GI symptom severity	High
7 (9%)	Constipation and bloating	Low

^1^: % is according to Black et al. [32]. Abbreviations: GI, gastrointestinal; IBS, Irritable Bowel Syndrome.

**Table 2:** Main herbal-based remedies proposed for IBS management: observed effects, bioactive compounds, and clinical evidence.

Herbal-Based Remedy	Observed Effects	Study Type	Bioactive Compounds	IBS Subtype	Refs.
Peppermint oil (*Mentha piperita*)	Smooth muscle relaxation due to blockade of Ca^2+^ channels, pain modulation via TRPM8 and TRPA1, anti-inflammatory, anti-microbial due to the modulation of the immune system, improvement of the patient’s global assessment, IBS symptom severity score and stool frequency score	Preclinical and Clinical	Menthol, limonene, menthofuran, a-pinene, b-pinene, neomenthol, isomenthone, 1,8-cineole	Not specified	[61,62,63,64,65,66,67,68,69,70]
Iberogast (STW 5)	Smooth muscle relaxation, gastric secretion stimulation, anti-inflammatory effect due to NF-κB, STAT1, and iNOS inhibition, improvement of tight junction disruption due to ZO-1 modulation, improvement of GI symptoms, IBS symptom severity score, and colonic gas tolerance in patients	Preclinical and Clinical	Phytochemicals from 9 plants including bitter candytuft, angelica root, milk thistle fruit, celandine herb, peppermint herb, caraway fruit, liquorice root, balm leaf, and chamomile flower	Not specified	[71,72,73,74,75,76]
Turmeric *(Curcuma longa)*	Anti-inflammatory and antioxidant effects, GI motility regulation via 5-HT modulation, improvement in gut microbiota alteration	In vivo animal studies	Curcumin	IBS-C (animal model)	[77,78,79,80]
Fennel essential oil (*Foeniculum vulgare*)	Smooth muscle relaxation due to K^+^ channel opening, anti-inflammatory effects, antispasmodic activity, reduction in abdominal pain and other IBS symptoms in patients with mild-to-moderate IBS, enhanced quality of life	Preclinical and Clinical	Anethole, fenchone, estragole, flavonoids	All patients’ subtypes	[81,82,83,84]
Ginger *(Zingiber officinale)*	Debate efficacy on IBS: spasmolytic and spasmogenic effects due to the inhibition of cholinergic response and K^+^-induced contractions, reduction in colonic edema and inflammation via NF-κB modulation, Decreased abdominal pain severity and frequency, bloating, depression, and anxiety scores in patients treated with an herbal mixture containing ginger	In vivo animal study and Clinical	Saponins, flavonoids, alkaloids, 6-gingerol	IBS-D (animal model)	[85,86,87,88]
Aloe *(Aloe vera)*	Laxative effect for IBS-induced constipation, increased intestinal motility and permeability, stimulation of mucus secretion, and release of prostaglandin-like compounds in the colon	Clinical	Barbaloin	IBS-D	[89,90,91]
Hemp *(Cannabis sativa)*	Modulation of gut motility, anti-inflammatory effects, improved gastric-distension pain and colitis recovery, pain reduction through action on CB_1_ and CB_2_ receptors and on TRP ion channels and PPAR-α/γ. In humans, improvement of multiple IBS symptoms (limited evidence)	Preclinical and Clinical	THC, CBD, CBG and β-caryophyllene	IBS-D, IBS-A, IBS-M	[92,93,94,95,96,97,98,99]
Coffee (*Coffea arabica* and other spp.)	Potential preventive effects, but may aggravate symptoms in diagnosed patients due to stimulation of intestinal motility, increased gastric secretion, and inflammation mediators	Observational	Caffeine, polyphenols, diterpenes, melanoidins	In women, overweight individuals and IBS-C patients may aggravate symptoms	[100,101,102,103]
Yellow gentian *(Gentiana lutea)*	Antispasmodic, stimulation of bile and digestive enzyme production	In vitro	Gentiopicroside, gentisin, swertiamarin	Not specified	[104]

Abbreviations: 5-HT (5-Hydroxytryptamine), CBD (cannabidiol), CBG (cannabigerol), GI (gastrointestinal), IBS (Irritable Bowel Syndrome), IBS-A (Irritable Bowel Syndrome—alternating subtype), IBS-C (Irritable Bowel Syndrome—constipation-predominant), IBS-D (Irritable Bowel Syndrome—diarrhea-predominant), IBS-M (Irritable Bowel Syndrome—mixed subtype), iNOS (inducible nitric oxide synthase), NF-κB (nuclear factor kappa-light-chain-enhancer of activated B cells), PPAR (peroxisome proliferator-activated receptor), STAT1 (signal transducer and activator of transcription 1), THC (tetrahydrocannabinol), TRPA1 (transient receptor potential ankyrin 1), TRPM8 (transient receptor potential melastatin 8), ZO1 (zonula occludens-1, tight junction protein 1).

**Table 3:** Main clinical studies assessing safety and efficacy of herbal-based remedies for IBS management.

Herbal-Based Remedy	Study Type	Study Duration	Number of Patients	Observed Efficacy and Adverse Effects	Refs./Clinical Trials’ ID
Peppermint oil (*Mentha piperita*)	Multi-center, open-label, single-arm, phase 3 trial	4 weeks (3 times a day before meals)	69 Japanese IBS patients (17–60 years of age)	Improved patients’ global assessment (PtGA) starting from week 2 Reduced IBS symptom severity Non-serious adverse effects in 20% of subjects	[70]
Iberogast (STW 5)	Prospective observational study	1 week	980 children (3–14 years of age)	Improved gastrointestinal symptom score (GIS) Good or very good tolerability for 95% of subjects	[75]
Iberogast (STW 5)	Meta-analysis of 5 clinical trials	4–8 weeks	900 patients	Improved GI symptoms vs. placebo	[112]
Iberogast (STW 5)	Cross-over, randomized, double-blind, placebo-controlled trial	2 weeks	10 patients	Improved colonic gas tolerance	[76]
Turmeric *(Curcuma longa)*	Meta-analysis on randomized controlled trials and comparative observational studies	4–18 weeks	551 adult IBS patients	Improved general quality of life Improved IBS score Reduced abdominal pain	[80]
Fennel essential oil (*Foeniculum vulgare*)	Randomized, double-blind, placebo-controlled trial	1 month	121 adult patients	Improved symptoms and quality of life Good tolerability and safety	[83]
Combination of turmeric and essential fennel oil	Observational, prospective, non-controlled, non-randomized study	2 months	211 adult patients	Improved symptoms and quality of life	[84]
Ginger *(Zingiber officinale)*	Double blind randomized controlled trial	4 weeks	45 adult patients	No difference with respect to placebo Well tolerated	[87]
Combination of *Boswellia carterii, Zingiber officinale*, and *Achillea millefolium*	Randomized controlled trial	3 months	60 adult patients	Improved symptoms Reduction in IBS-related anxiety and depression	[88]
Aloe *(Aloe vera)*	Meta-analysis on randomized controlled trials	1–3 months	151 patients	Improved IBS symptom score No difference in adverse events vs. placebo	[89]
Aloe *(Aloe vera)*	Post hoc analysis of two randomized double-blind controlled studies	4 weeks	213 patients	Improved symptoms in IBS-D patients Safe and well tolerated	[91]
Cannabidiol chewing gum	Randomized, double-blinded, placebo-controlled cross-over trial	8 weeks	32 female patients	No significant difference in pain scores vs. placebo High intra- and inter-individual variation	[98]
Bedrocan^®^ cannabis treatment	Prospective study	6 months	56 fibromyalgia patients with dyspepsia and/or IBS	Improved IBS symptoms	[99]
Coffee (*Coffea arabica* and other spp.)	Meta-analysis	regular coffee consumption	432,022 patients	Potential preventive effects and reduced likelihood of IBS development	[100]
Coffee (*Coffea arabica* and other spp.)	Observational study	regular coffee consumption	99 healthy subjects	Increased motility index within four minutes after coffee consumption	[103]
Coffee (*Coffea arabica* and other spp.)	Meta-analysis	regular coffee consumption	3362 Iranian adults	Association between regular coffee consumption and IBS	[102]
Tong-Xie-Yao-Fang	Double-blind, placebo-controlled randomized trial	4 weeks	155 patients	Improved symptoms in IBS-D patients	[108]
Chinese herbal medicine	Meta-analysis of 10 randomized controlled trials	4–8 weeks	2501 adult patients with IBS-D	Improved IBS symptoms Higher rate of adverse events vs. placebo	[107]

Abbreviations: CBD (cannabidiol), GI (gastrointestinal), GIS (gastrointestinal symptom score), IBS (Irritable Bowel Syndrome), IBS-A (Irritable Bowel Syndrome—alternating subtype), IBS-C (Irritable Bowel Syndrome—constipation-predominant), IBS-D (Irritable Bowel Syndrome—diarrhea-predominant), IBS-M (Irritable Bowel Syndrome—mixed subtype), PtGA (patient’s global assessment), RCT (randomized controlled trial), STW 5 (standardized herbal extract 5-Iberogast).
